# On the Protein Fibrillation Pathway: Oligomer Intermediates Detection Using ATR-FTIR Spectroscopy

**DOI:** 10.3390/molecules26040970

**Published:** 2021-02-12

**Authors:** Jelica Milošević, Radivoje Prodanović, Natalija Polović

**Affiliations:** Department of Biochemistry, Faculty of Chemistry, University of Belgrade, Studentski trg 12, 11000 Belgrade, Serbia; jelica@chem.bg.ac.rs (J.M.); rprodano@chem.bg.ac.rs (R.P.)

**Keywords:** ATR FTIR, oligomer intermediates, amyloid fibrillation, HEWL, secondary structure perturbation

## Abstract

Oligomeric intermediates on the pathway of amyloid fibrillation are suspected as the main cytotoxins responsible for amyloid-related pathogenicity. As they appear to be a part of the lag phase of amyloid fibrillation when analyzed using standard methods such as Thioflavin T (ThT) fluorescence, a more sensitive method is needed for their detection. Here we apply Fourier transform infrared spectroscopy (FTIR) in attenuated total reflectance (ATR) mode for fast and cheap analysis of destabilized hen-egg-white lysozyme solution and detection of oligomer intermediates of amyloid fibrillation. Standard methods of protein aggregation analysis— Thioflavin T (ThT) fluorescence, atomic force microscopy (AFM), and 8-anilinonaphthalene-1-sulphonic acid (ANS) fluorescence were applied and compared to FTIR spectroscopy data. Results show the great potential of FTIR for both, qualitative and quantitative monitoring of oligomer formation based on the secondary structure changes. While oligomer intermediates do not induce significant changes in ThT fluorescence, their secondary structure changes were very prominent. Normalization of specific Amide I region peak intensities by using Amide II peak intensity as an internal standard provides an opportunity to use FTIR spectroscopy for both qualitative and quantitative analysis of biological samples and detection of potentially toxic oligomers, as well as for screening of efficiency of fibrillation procedures.

## 1. Introduction

Amyloid fibrils are highly ordered protein aggregates with monotonous cross-β structure independent of the sequence [1]. Proteins occupy different intermediate states upon their amyloid fibrillation. These states show diverse degrees of stability as they represent local minima on the way to ultimate stabilization in the form of amyloid fibrils [2]. In the first, lag stage of amyloid misfolding, the native state of a protein is destabilized and transformed into nuclei for fibrillation. Once formed they can proceed to metastable oligomers which are considered the actual toxic form inducing misfolding-related pathological states [3]. There is a discrepancy in the use of the term oligomers. A certain group of authors takes some forms of protofibrils as oligomeric intermediates, while others reserve this term for spherical intermediates that precede fibrillar forms. The widest definition takes oligomers as smaller aggregates containing 2–20 monomer units [1]. Thus, oligomer is used as a term for a state that excludes 50% of protein molecules in a monomer state and the other case where more than 50% is part of a big cluster [4]. They continue their route to fibrils by occupying protofibril form—wormlike filamentous late intermediate stage without periodicity in their structure [5,6]. Protofibrils assemble into protofilaments which represent single-stranded fibrils that twist helically to form mature fibrils.

As oligomers are proved to be toxic species either when in vitro formed [7], or in vivo formed in many misfolding diseases [8], their detection is of high importance. Unlike fibrils that grow very fast once their structural form is constructed, oligomers grow slowly [1]. Depending on the starting protein characteristics and fibrillation procedure, the time scale of this process varies from hours to weeks. Lysozyme from chicken egg white in the high-ethanol condition is an example of a slow fibrillating model system [9] suitable for oligomer detection and analysis.

Lysozyme (3.2.1.17) is a diverse group of antimicrobial enzymes with N-acetylmuramide glycanhydrolase activity. By catalyzing the hydrolysis of 1,4-beta-linkages between N-acetylmuramic acid and N-acetyl-D-glucosamine, it protects animals from gram-positive bacteria [10]. Hen egg-white lysozyme (HEWL) is serving its protective role during chick embryonic development. It is a globular protein with only 14.3 kDa molecular weight. The secondary structure content of native HEWL is described as mostly α-helicoidal (about 40%) with a significant part of unordered regions and the presence of a small β-sheet (12%) [10,11]. A unique property of HEWL is its extremely high pI value due to 8% of arginine among amino acid residues. This protein can be destabilized in many different ways, so there are a variety of different protocols for its aggregation in both, non-ordered and ordered form. Nevertheless, it appears to be quite soluble even when it occupies different nonnative forms, which increases its propensity for directing misfolding intermediates toward ultimate stabilization in the form of amyloid fibrils. Conditions favoring amyloid state vary from extremely acidic (about pH 2) [12,13,14] common for many different proteins, to extremely alkaline of pH above 12 [15,16,17]. Among different fibrillation protocols are incubations in the presence of high concentrations of ethanol or chaotropic agents [18]. Nonnative forms of this protein can stay soluble in a high ethanol concentration of about 90% [19], while most proteins get quantitatively precipitated in ethanol higher than 60% regardless of their conformation. Macroscopic precipitation of HEWL in 90% ethanol does not take place even in a high protein concentration of about 6 mg/mL for days.

Among standard techniques for amyloid fibrillation monitoring, the highest application goes to spectroscopy techniques based on the properties of specific fluorescent dyes that interact with a cross-β-sheet structure—Thioflavin T (ThT) and Congo Red. Microscopy techniques are also useful, especially in determining fibril length, together with dynamic light scattering (DLS). As the amyloid state is characterized by uniform β-sheet structure, secondary structure analysis by circular dichroism (CD) or infrared (IR) spectroscopy also plays a significant role in fibrillation monitoring. Infrared spectroscopy has a very high potential for the analysis of protein aggregation, including amyloid forms [20,21] as it is more suitable than CD for turbid samples. It was previously applied for the analysis of different stages of amyloid formation [21,22]. Deconvolution of the Amide I region provides the exact determination of secondary structure content [23] but requires a lot of operations in the spectral processing so it has never acquired wide application for numerous sample analyses, such as biological samples for oligomer presence screening [24]. We have previously published a study introducing a simple and fast method for mature fibrils detection based on attenuated total reflectance—Fourier transform infrared spectroscopy (ATR-FTIR) [25]. The method eliminates complicated spectra processing and offers reliable fibrillation monitoring by only calculating the ratio of characteristic Amide I band intensities and Amide II maximum intensity. Our aim here was to examine the application of ATR-FTIR spectroscopy and this simple method for spectral analysis for the monitoring of misfolding oligomer formation. This aim was set as the standard method for amyloid monitoring—ThT fluorescence is not sensitive enough for this application due to poor interactions between oligomer states and the dye [26]. The toxicity of the oligomeric state makes it highly important to establish a sensitive, fast, and cheap method for their detection, as oligomers are targets for potential treatments of neurodegenerative misfolding diseases that are under extensive investigation.

## 2. Results and Discussion

In order to study protein structural transitions that precede fibrillation using ATR-FTIR, we chose HEWL as a model protein for slow fibrillation. Many studies are published regarding HEWL amyloid fibrillation—protocols and analysis of fibrils formed. It appears that HEWL is an extraordinary protein with increased solubility in various (destabilizing) conditions. Prolonged solubility in the presence of a very high percentage of organic solvents such as ethanol favors slow and organized aggregation in the form of amyloid fibrils [9]. As a result of its high availability and high propensity to amyloid and amyloid-like states, HEWL proved to be among proteins with great importance for establishing a new methodology regarding amyloid formation monitoring, including analysis of intermediate fibrillation forms [21,22].

**Scheme 1:**
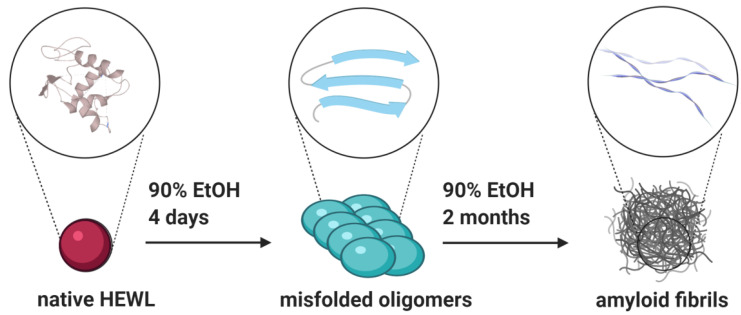
Schematic representation of hen egg-white lysozyme (HEWL) fibrillation in 90% ethanol solution.

We incubated HEWL in conditions favoring amyloid formation—90% ethanol, 6 mg/mL protein concentration at room temperature [19,27,28]. It was previously shown that fibrillation in high ethanol conditions is a nucleation-dependent polymerization pathway characterized by a sigmoidal curve with an initial lag phase [9]. Different studies report a long fibrillation process in high-ethanol conditions taking over a week at room temperature [28]. Based on the different methodology applied, they propose various timescale of the fibrillation process, but many papers agree that the complete formation of long mature fibrils, detectable by sophisticated microscopy techniques, takes one to two months [9,27]. Holley and coworkers reported the aggregation kinetics study where the initial lag phase took between five and nine days for individual experiments [9] while the formation of long mature fibrils that occupy the upper plateau of the sigmoidal curve took about 30 days. Scheme 1 is based on these previous studies [9,19,27,28] that have described carefully in much detail HEWL fibrillation dynamics, supporting the formation of oligomers after four days’ incubation period and completely mature fibrils after 60 days. It shows the representation of HEWL fibrillation kinetics under conditions that we applied for the design of our experiments focused on oligomer analysis by ATR-FTIR.

### 2.1. Formation of Oligomers: ThT and ANS Fluorescence

**Figure 1:**
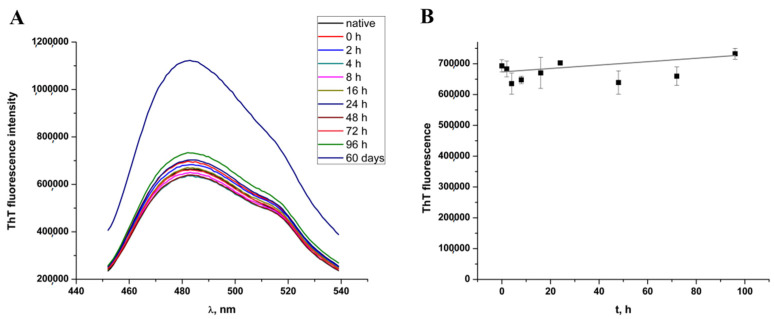
(**A**) Thioflavin T (ThT) fluorescence spectra during four day’s HEWL incubation in 90% ethanol and after a 60-day incubation period. (**B**) Time-dependent change of ThT fluorescence over four days’ incubation of HEWL in 90% ethanol solution.

The formation of oligomers is monitored by measuring ThT binding and hydrophobic region exposure. Figure 1A shows ThT emission spectra of completely native HEWL dissolved in 100 mM Tris-HCl buffer of pH 8 and samples obtained during the first four days of incubation in 90% ethanol solution. For comparison, the ThT fluorescence of a mature amyloid fibril obtained after 60-day incubation is presented as well. Within the first four days, a moderate increase in ThT fluorescence was detected. To show that differences in ThT fluorescence between the samples incubated for four days in the presence of ethanol are not significant and all correspond to the lag phase, we present ThT fluorescence dependence on the incubation time (Figure 1B). The data shown are based on the fluorescence intensity at the maximum of emission spectra (484 nm). A large increase of ThT fluorescence was detected after two months following complete HEWL fibrillation. The detected changes in ThT fluorescence are in concordance with previously published results using the same methodology [9], as well as literature data about HEWL fibrillation based on analog techniques [9,28].

**Figure 2:**
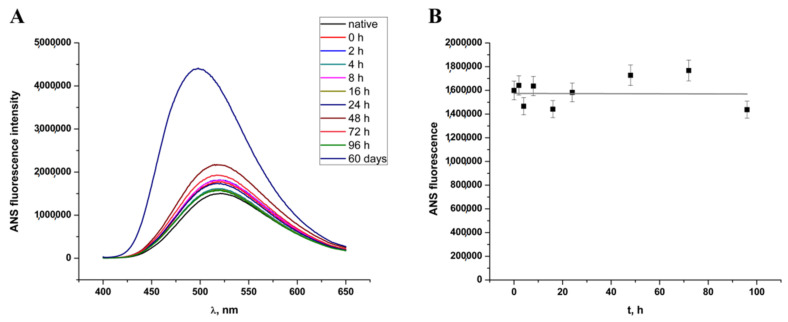
(**A**) 8-anilinonaphthalene-1-sulphonic acid (ANS) fluorescence spectra during four days’ HEWL incubation in 90% ethanol and after a 60-day incubation period. (**B**) Time-dependent change of ANS fluorescence during four days’ incubation of HEWL in 90% ethanol solution.

To get an insight into the exposure of hydrophobic regions, we monitored 8-anilinonaphthalene-1-sulphonic acid (ANS) fluorescence as well, and similar trends were observed (Figure 2A,B). Spectra gathered during the first four days of incubation does not show significant change while prolonged incubation in a high concentration of ethanol results in a more than a twofold increase of the total ANS fluorescence intensity indicating higher exposure of the hydrophobic patches. When presented as time-dependent ANS fluorescence based on emission maximum at 500 nm (Figure 2B), results also indicate a good correlation with ThT fluorescence data showing that HEWL stays in a lag phase over the time course of 4 days. The correlation of ThT and ANS fluorescence trends is not surprising taking into account fibrillation conditions of decreased solvent polarity that prevent hydrophobic collapse for additional stabilization of fibrils.

### 2.2. Oligomer Morphology: Atomic Force Microscopy (AFM)

**Figure 3:**
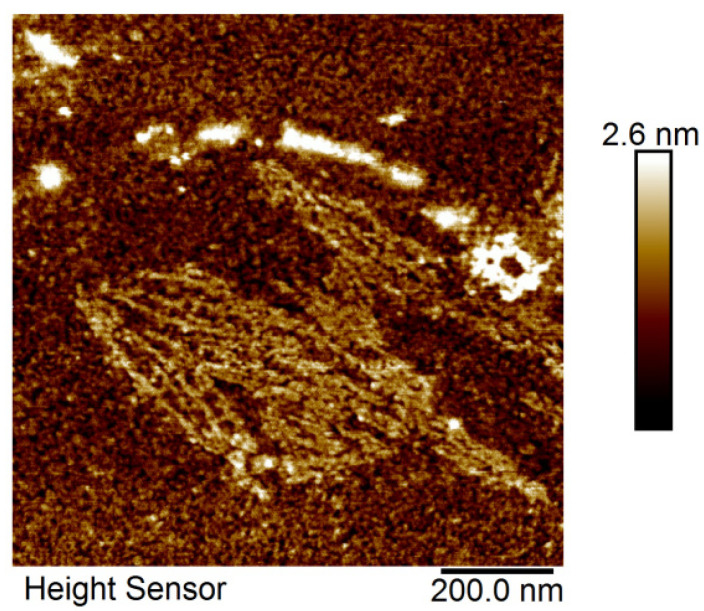
AFM microscopy of HEWL oligomer intermediates.

In order to get an insight into the morphology and structure of the HEWL state formed during the lag phase of incubation, we applied AFM microscopy. It is a suitable method for discrimination between the oligomer and other protein states on the fibrillation pathway [24,29]. After four days of incubation, there is a heterogeneous population of HEWL states mostly including misfolded oligomers but also some short fibrils as captured together in the scan presented in Figure 3. Rare forms of 1–3 nm width indicate a slow transition to further states of fibrillation, but most of the molecules are part of the smaller thin fibrillar forms that can be attributed to misfolded oligomer intermediates [22]. These aggregates do not appear to be fully grown amyloid fibrils according to their length and diameter estimated based on the AFM, as mature fibrils require at least 100 nm length and 10 nm diameter [26]. Even though the oligomer-specific antibodies were not applied here, the morphology of aggregates formed during four-day incubation suggests it is an oligomeric state shown also in the case of ataxin and named misfolded oligomers [24]. This result is in concordance with the dynamics of amyloid formation published previously and discussed above [9].

### 2.3. Qualitative Analysis of FTIR Spectra: Amide I Region

For structural analysis of these intermediate states, we applied ATR-FTIR. Due to the conformation dependence of infrared light absorption, amide regions of infrared spectra provide a useful tool for both qualitative and quantitative analysis of protein secondary structures.

**Figure 4:**
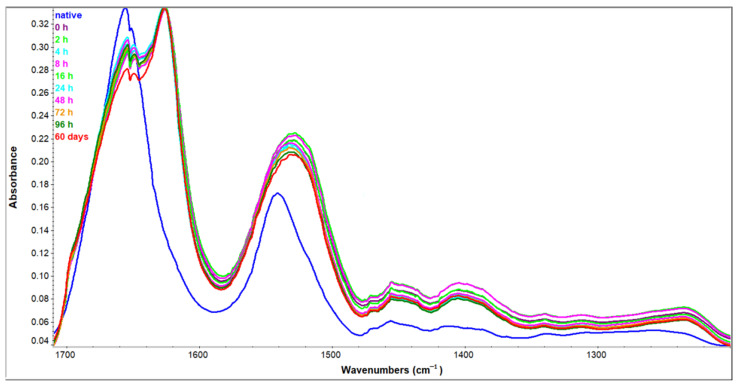
Amide I—Amide III regions of HEWL ATR-FTIR spectra. Native—HEWL in H_2_O, 0 h–60 days—HEWL samples incubated in 90% C_2_H_5_OH for the time period indicated.

Figure 4 shows the three most important amide regions for protein structural analysis—Amide I, Amide II, and Amide III region spanning from 1700 to 1200 cm^−1^. Spectra presented in the figure include a sample of native HEWL in H_2_O solution, as well as samples incubated in 90% ethanol (C_2_H_5_OH) solution for the time period indicated in the figure legend (up to four days and 60 days for comparison to fully amyloid state). Even a brief inspection of spectra suggests significant perturbations at the higher wavenumbers. Those are the changes in the Amide I region (1600–1700 cm^−1^), the one with the highest conformational dependence and sensitivity among all amide regions. Changes in the adjacent Amide II region (1600–1500 cm^−1^) appear to be less dependent on the secondary structure content and are the consequence of the drastic solvent change. In order to explore the complete potential of ATR-FTIR spectra, individual Amide regions were analyzed separately.

**Figure 5:**
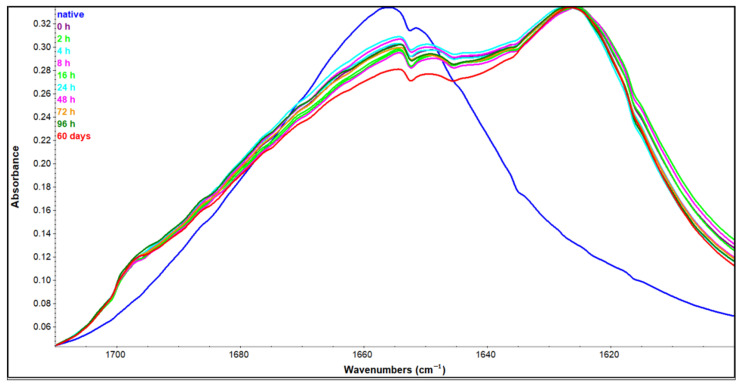
Amide I region of native HEWL sample in H_2_O solution and HEWL samples incubated in 90% C_2_H_5_OH for the time period indicated.

Amide I region arises mainly from C = O stretching vibrations and out-of-phase CN stretching vibrations of polypeptide backbone [30]. Between 1600–1700 cm^−1^ each secondary structure contributes to the absorption in a certain wavenumber range. Upon amyloid fibrillation, due to prominent secondary structure change, spectral differences are very pronounced. The most characteristic band of amyloid IR spectra has a maximum in the range from 1611 cm^−1^ [31] to 1628 cm^−1^ [32] depending on the (poly)peptide sequence and fibrillation conditions [33]. These maxima arise on the count of nativelike secondary structure peaks in the starting protein sample. Figure 5 shows all the spectra—native HEWL in H_2_O, and those incubated in 90% ethanol solution in the Amide I region. The spectrum of native HEWL shows prominent bands at about 1650 cm^−1^ corresponding to α-helix and random coil, and a less prominent band at 1634 cm^−1^ corresponding to intramolecular β-sheet [20]. The transition of HEWL to 90% ethanol solution induced significant spectral changes. There is a redshift accompanied by disproportionation of particular bands leading to completely different spectra. The intermolecular β-sheet bands at about 1620 cm^−1^ and 1700 cm^−1^ arise drastically while secondary structures present in the native HEWL, including α-helix and random coil, decrease. This decrease is not complete as a certain pool of protein still retains these secondary structures. Nevertheless, an increase in low-frequency (about 1620 cm^−1^) [22] and, to a lesser extent, high frequency (1700 cm^−1^) [25] β-sheet band is indicative of amyloid pathway intermediates. The first spectrum obtained immediately after the transition of HEWL to ethanol-rich conditions has these characteristic features and they do not seem to change significantly over time. Spectra collected for the samples incubated in the period of zero to four days (0 to 96 h in the figure legend) at increased ethanol concentration are quite overlapped in the Amide I region. Even the sample incubated for 60 days does not seem to change extremely, but it shows a further drop in native-like secondary structures that is not negligible. This indicates that almost all secondary structure changes take place immediately after stressing HEWL by the lowered polarity of the solution, suggesting that monitoring of oligomer formation using FTIR could be more sensitive than ThT fluorescence. 

Oligomer to fibril transition during HEWL acid fibrillation was monitored by Zou et al. [23]. In those conditions, they also acquired spectra with a similar overall shape for oligomers and fibrils, but they found out a slight redshift from 1622 cm^−1^ to 1618 cm^−1^ for aggregation β-sheet band also reported in other researches [34]. In the case of ethanol-induced fibrillation, such change was not detected suggesting that almost complete rearrangement of the secondary structure takes place during the lag phase.

**Figure 6:**
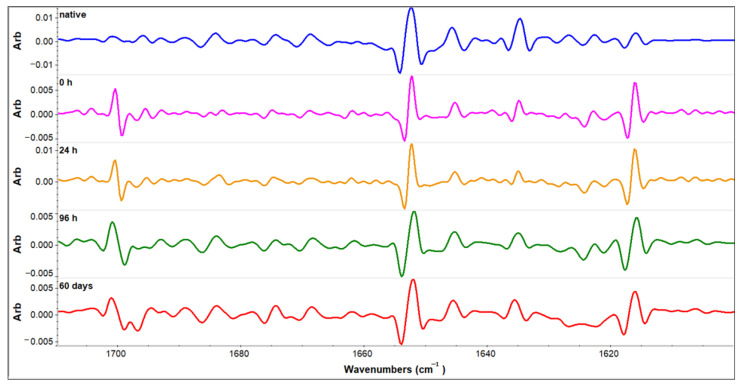
Second derivative spectra of native HEWL sample in H_2_O solution and HEWL samples incubated in 90% C_2_H_5_OH for the time period indicated.

For more in-depth analysis of this region, second derivative spectra are calculated for the native sample in an aqueous solution, starting sample in ethanol solution (0 h), intermediates incubated one and four days in the ethanol solution, and the amyloid sample obtained by 60 days’ incubation in the same conditions. The second derivative spectra are presented in Figure 6. They show more clearly that a slight decrease in the α-helix band takes place (1654 cm^−1^), together with a random coil decrease (1644 cm^−1^) giving rise to an aggregation-specific band at 1618 cm^−1^ [20,22,25]. To get a quantitative assessment of these changes, the correlation coefficient was calculated to compare the initial spectrum of native HEWL and the other four spectra obtained for the samples incubated—0 h, 24 h, 96 h, and 60 days in 90% ethanol. Correlation coefficients for these incubation periods are 0.68, 0.68, 0.65 and 0.64, respectively. These results prove that the most significant level of secondary structure changes takes place in the short time frame upon solvent shift, and further secondary structure changes are slow.

### 2.4. Qualitative Analysis of FTIR Spectra: Amide II Region

**Figure 7:**
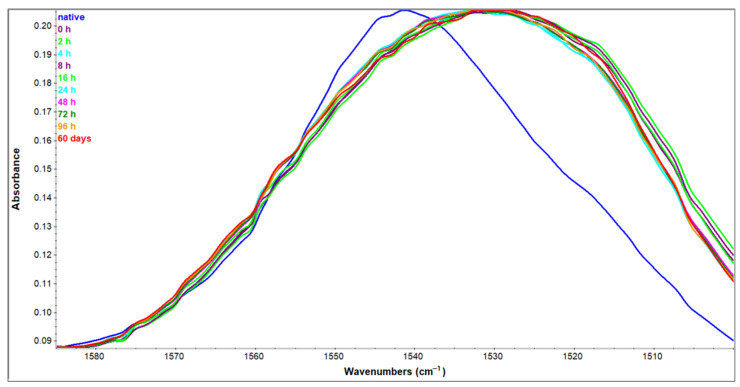
Amide II region of HEWL native sample in H_2_O solution and HEWL samples incubated in 90% C_2_H_5_OH for the time period indicated.

Regardless of complete perturbations in the Amide I region, HEWL spectra in the Amide II region show only a slight shift of spectral maximum (from 1550 cm^−1^ to 1540 cm^−1^) due to transition to less polar conditions (from H_2_O to 90% ethanol solution) (Figure 7). This change in maximum position of about 10 cm^−1^ is far less than a change characteristic for H-D exchange that results in a shift of about 100 cm^−1^ [35]. The Amide II region arising from NH in-plane bending and CN stretching is far less dependent on specific secondary structures presence than the Amide I region [30,36]. The low sensitivity to secondary structure change makes this region suitable for use as an internal standard for comparison of Amide I band intensities of different samples [25,37,38,39].

### 2.5. Qualitative Analysis of FTIR Spectra: Amide III Region

**Figure 8:**
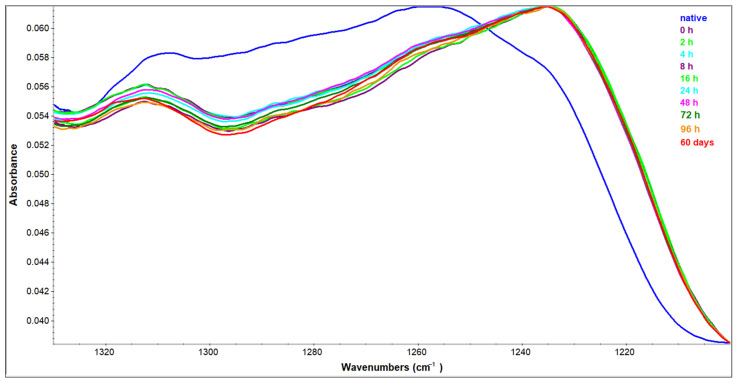
Amide III region of HEWL native sample in H_2_O solution and HEWL samples incubated in 90% C_2_H_5_OH for the time period indicated.

The Amide III region is generally regarded as a low-sensitive region of the protein IR spectrum. The main contribution to its bands comes from the NH bending and the CN stretching vibrations which appear to be conformational dependent [30]. Structural transition on the way to amyloid fibrils results in prominent changes in this region as well. Spectra presented in Figure 8 are analyzed according to ranges of Amide III band positions examined thoroughly by Cai and Singh [40,41]. Native HEWL has peaks that can unambiguously be attributed to α-helix (1310 cm^−1^), random coil (1257 cm^−1^), and β-sheet (1236 cm^−1^). Along with the change of conditions, the spectrum is transforming into a β-sheet rich one. Judged by the Amide III region, it seems that the increase in β-sheet content takes place on the count of a random coil and α-helix. Even though quite illustrative, the Amide III region cannot distinguish between intra- and intermolecular β-sheet as can be differentiated in the Amide I region.

### 2.6. Quantitative Analysis of FTIR Spectra: Amide I Region Deconvolution

As even simple inspection of the Amide I region suggests prominent secondary structure changes during the first four days of incubation in the ethanol solution, and thus the fact that FTIR is promising for oligomer detection, we wanted to use it quantitatively by determining the secondary structure contents. Deconvolution of the Amide I region of FTIR spectra is a standard method for secondary structure analysis with great application among different protein model systems [20,23,42]. This demanding procedure was applied on selected samples including spectra of native, water dissolved HEWL and 0 h, 24 h, 96 h, and 60-days incubated samples in ethanol-rich conditions. The Amide I region of each spectrum was decomposed to its original Gaussian constituents that are attributed to certain secondary structures according to previously published data [20,21,23,25,43,44,45]. The area under Gaussian constituents was calculated and expressed as a percentage of the total summed area. This way, the contribution of each constituent peak to the total spectrum, and thus—the content of each secondary structure in percentage was determined. Results presented in Table 1 are compared to X-ray diffraction determined secondary structure content and the results show a great correlation between these literature data and data obtained for native HEWL. The dominant secondary structure in native HEWL is α-helix with 41.7% (42.6% from X-ray data) and it drops to about 20% after the transition to ethanol. A further drop in α-helix content is in the error range so it suggests that a great part of α-helix is highly destabilized at the moment of transition to 90% ethanol. Native-like β-sheet content remains about 10% regardless of the condition change and incubation time. Total β-sheet content of the native HEWL determined by deconvolution of FTIR spectrum does not differ from the total β-sheet content obtained by X-ray diffraction. In other samples, aggregation-specific β-sheet increases, and this change is the most significant upon transition to ethanol (from 8.6 to 29.7%). The change is still prominent after the first incubation day (34.7%), but it gets much slower in the next three days indicating a stable oligomer state. The final transition of the oligomer to mature fibrils gives an increase in the aggregation-specific β-sheet to 38.2%. As α-helix and intramolecular β-sheet does not change significantly from 0 h to 60 days of incubation, this increase in aggregation-specific β-sheet comes mostly from a more gradual decrease in the content of random coil and turn presented together here.

Almost the same changes in the content of secondary structures obtained by deconvolution of FTIR spectra were observed in heat- and acid-induced HEWL fibrillation [20]. As in their case, the change between native and 60-day incubated HEWL includes about a 30% increase of aggregation-specific β-sheet, random coil drop of up to 10%, and α-helix loss from 40% to 20% [20].

In ethanol-rich conditions, CD spectroscopy was applied for secondary structure content monitoring by Goda et al. [28]. They incubated HEWL for 24 h and the overall change of β-sheet content (about 20%) is similar to our data (26%) for the same incubation period. Even though the overall change was similar, CD spectroscopy data did not provide a good correlation of the native sample with the standard structural methods such as X-ray diffraction and synchrotron radiation circular dichroism [10,11] as was the case with FTIR deconvolution data presented here.

### 2.7. Quantitative Analysis of FTIR Spectra: Amide I/AMIDE II Ratio

**Figure 9:**
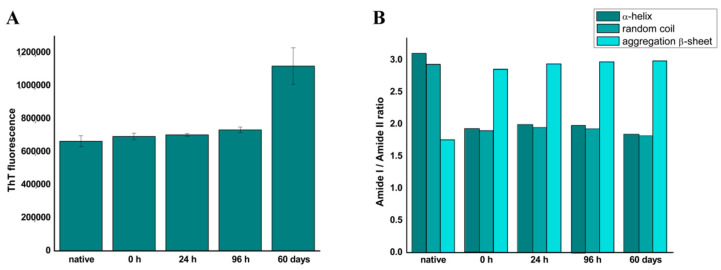
(**A**) ThT fluorescence and (**B**) Amide I/Amide II ratio for native, 0 h-, 24 h-, 96 h-, and 60-day-incubated HEWL sample in 90% ethanol. Bands were attributed to the secondary structures as follows: 1654 cm*^−^*^1^ to the α-helix, 1644 cm*^−^*^1^ to the random coil, 1620 cm*^−^*^1,^ and 1698 cm^−1^ to the aggregation-specific β-sheet, 1542 and 1534 cm^−1^ to the Amide II internal standards.

As deconvolution is a demanding method unsuitable for big sets of samples, we wanted to test another, much easier and faster method for the quantitative analysis of FTIR spectra. We had previously published a simple method for the quantitative detection of amyloid fibrils by monitoring of high- and low-frequency aggregation-specific β-sheet in the Amide I region [25]. It was shown that the increase of these bands intensities when normalized to the Amide II maxima intensity can be used as a parameter in quantitative monitoring of amyloid formation for a fast-fibrillating model system ovalbumin [25]. This normalization can be done by calculating Amide I to Amide II ratios, namely by dividing the value of absorbance of the sample at the wavenumber of interest in the Amide I region, with the absorbance at the Amide II maximum. Bands of interest here were at the following wavenumbers: 1654 cm*^−^*^1^ for α-helix, 1644 cm*^−^*^1^ for random coil, 1620 and 1698 cm*^−^*^1^ for aggregation-specific β-sheets. Once Amide I maxima were identified, their intensities were divided with the intensity of the Amide II maximum at 1542 cm*^−^*^1^ for the sample dissolved in H_2_O and 1534 cm*^−^*^1^ for samples in C_2_H_5_OH. Calculation of these ratios in all samples of interest resulted in the values that indicate a trend of secondary structure change in a process observed. These values do not show secondary structure contents but give reliable information about the propensity of a sample to occupy a certain secondary structure [25,37,38,39]. The increase of aggregation-specific β-sheet monitored this way was found to be in a great correlation with the ThT fluorescence increase during the amyloid formation of a fast-fibrillating model protein [25]. The samples of our model system for slow-fibrillating proteins—HEWL was the subject of similar analysis and the results are presented in Figure 9B. The same samples analyzed by the standard quantitative analysis method—deconvolution of the Amide I region, were analyzed by this method as well.

From the data presented in Figure 9, it is clear that Amide I/Amide II intensities ratio provides better insight into oligomers formation than ThT fluorescence data which is not surprising as ThT is characterized as a fluorescent dye with low affinity for the oligomer state [26]. Sum of normalized intensities of peaks attributed to aggregation specific intermolecular β-sheets (1622 cm^−1^ and 1698 cm^−1^) increases strongly upon the formation of oligomers (4 days) and continues to increase during further fibrillation (60 days). At the same time, the propensity to form nativelike structures (α-helix and random coil) decreases. The overall data show an almost immediate transition from native to the oligomer structure.

Superior detection of oligomers by applying normalized intensities of Amide I bands to ThT fluorescence is also shown as a quite poor correlation between these two data sets (Supplementary Figure S1). It thus proves that FTIR spectroscopy and simple normalization of the intensity of aggregation specific β-sheet bands gives undoubted proof of oligomer formation.

## 3. Materials and Methods

### 3.1. Materials

Lysozyme from chicken egg white, Thioflavin T (ThT), 8-anilinonaphthalene-1-sulphonic acid (ANS) were purchased from Sigma–Aldrich (Steinheim, Germany). Coomassie brilliant blue R-250 (CBB R-250) was purchased from Serva (Heidelberg, Germany). Unstained protein molecular weight markers were purchased from Thermo Scientific (Rockford, IL, USA). All other chemicals used were analytical grade commercial products used without further purification.

### 3.2. HEWL Destabilization and Incubation

Commercial HEWL powder was dissolved in MiliQ water to the concentration of 60 mg/mL and further diluted by the addition of absolute ethanol to the final concentration of 90% (v/v) resulting in a protein concentration of 6 mg/mL. The solution was incubated at 20 °C with gentle shaking. Aliquots were removed at specific time points during the four-day-long incubation (0 h, 1 h, 2 h, 4 h, 8 h, 16 h, 24 h, 48 h, 72 h, 96 h) and after 60 days, and analyzed using biochemical and biophysical techniques.

### 3.3. ThT Fluorescence

ThT and buffer were mixed with HEWL samples including native protein dissolved in H_2_O, and aliquots taken during incubation in 90% ethanol solution (0 h, 1 h, 2 h, 4 h, 8 h, 16 h, 24 h, 48 h, 72 h, 96 h, 60 days) before spectra collection. The mixture contained 200 μL of ThT solution of 100 μM concentration, 1800 μL of 100 mM Tris-HCl buffer pH 8, and 200 μL of HEWL samples prediluted to 0.6 mg/mL in the same buffer. The excitation wavelength was 440 nm, and emission spectra were collected in the range 450 to 550 nm using Spectrofluorimeter FluoroMax-4 Jobin Yvon. Spectra were corrected for background signal contribution and multiplied with the dilution factor. All spectra were collected in triplicates and the results presented are the average of all three measurements. Emission maximum at 484 nm was used for the construction of time-dependence diagrams.

### 3.4. ANS Fluorescence

Mixtures containing 200 μL of 8 mM ANS, 1900 μL of 100 mM Tris-HCl buffer pH 8, and 100 μL of HEWL samples prediluted to 0.6 mg/mL in the same buffer were prepared. All emission spectra in the range of 400 to 600 nm were collected after the excitation with 390 nm wavelength. All samples (native HEWL in H_2_O, and samples incubated in the presence of ethanol) were treated the same way with triple spectra collected for each. Spectra were collected using Spectrofluorimeter FluoroMax-4 Jobin Yvon and background correction was applied. Fluorescence intensities were multiplied with the dilution factor. Emission maximum at 500 nm was used for the construction of time-dependence diagrams.

### 3.5. Atomic Force Microscopy

A sample of four-day incubated HEWL was scanned using AFM in tapping-mode (BioScope Resolve, Bruker, Germany). Before scanning, the sample was diluted to a concentration of 10 μg/mL and was applied onto Mica disc (model SD-101, Bruker, Germany), rinsed with water, and dried by the inert gas stream. Images were captured and analyzed using Nanoscope 8.10 software.

### 3.6. Fourier Transform Infrared Spectroscopy (FTIR)

Infrared spectra of HEWL samples (in H_2_O and C_2_H_5_OH) were collected using Nicolet Summit FTIR Spectrometer (Thermo Fisher Scientific) in ATR mode. Small aliquots of 1.5 μL in protein concentration 6 mg/mL were applied onto a diamond crystal and solvent was evaporated in the stream of argon. Composite spectra of the mid-IR region (400-4000 cm^−1^) were collected in 64 scans using the DTGS KBr detector. Spectra were automatically corrected for the background absorption. OMNIC software was used for two basic additional corrections—automatic ATR correction and baseline correction.

### 3.7. Spectral Analysis: Second Derivatives and Spectral Correlation

FTIR spectra were analyzed in the Amide I, Amide II, and Amide III regions. Spectra in the Amide I region were derived using the Savitsky–Golay second derivative with seven points and polynomial order 3. For a quantitative comparison of the spectra, the correlation coefficient was calculated from second derivatives using Equation (1):

(1) $$r=\;\frac{\sum^{\;}x_{i}y_{i}}{\sqrt{\sum^{\;}x_{i}^{2}\;\sum^{\;}y_{i}^{2}}}$$

where *x* and *y* are absorbances of two spectra compared at each frequency point (*i*).

### 3.8. Spectral Analysis: Deconvolution of the Amide I Region

For a more detailed analysis of spectral changes, FTIR spectra of HEWL samples in the Amide I region were deconvoluted. This was applied on the native HEWL sample in H_2_O solution, and HEWL samples incubated 1, 4, and 60 days in 90% ethanol solution. The Amide I region was decomposed to its original Gaussian constituents by automatic peak resolve function in OMNIC software. Decomposed peaks were attributed to characteristic secondary structures according to literature data regarding deconvolution of spectra of HEWL in H_2_O solution [20], HEWL monitored by H-D exchange [35,43,44], homologous proteins [21], and band assignment in other proteins and their fibrils [23,25,45]. Bands were assigned as follows: 1616–1622 cm^−1^ aggregation-specific β-sheet, 1626–1640 cm^−1^ intramolecular β-sheet, 1641–1645 cm^−1^ random coil, 1650–1660 cm^−1^ α-helix, 1660–1680 cm^−1^ turns and unordered structures, 1690–1700 cm^−1^ aggregation-specific β-sheet. The area under each peak was determined and the total area was calculated as the sum of individual peak areas. Content of each secondary structure is given as a percentage of the area under the certain peak in the total area of the Amide I region calculated. Results were compared with HEWL secondary structure content determined by X-ray diffraction calculated from UniProt entry [https://www.uniprot.org/uniprot/P00698 (accessed on 24 December 2020)].

### 3.9. Spectral Analysis: Amide I/Amide II Band Ratio

Intensities of characteristic peaks within the Amide I region corresponding to specific secondary structures were normalized and compared between samples of native HEWL in H_2_O, and HEWL incubated for one, four, and 60 days in 90% C_2_H_5_OH. Bands corresponding to α-helix (1644 cm^−1^), random coil (1654 cm^−1^), and aggregation specific β-sheets (1620 and 1698 cm^−1^) were identified, and their intensities for each sample were normalized by dividing with the intensity of the Amide II maximum (at 1542 cm^−1^ in H_2_O and 1534 cm^−1^ in the case of 90% C_2_H_5_OH in H_2_O) intensity. Normalized values are thus Amide I/Amide II ratios for characteristic peaks and they are presented as dimensionless values suggesting overall trends in the secondary structure change upon condition switch from water solution to high-ethanol solution and prolonged incubation. Normalized values of two aggregation-specific β-sheet bands were summed.

## 4. Conclusions

The analysis and detection of oligomeric amyloid intermediates are of crucial importance as these are the cytotoxic agent on the road to amyloid fibrils. HEWL was chosen as a model system for this sort of investigation and the establishing of FTIR methodology for oligomer detection in this study. Stress, induced by a strong decrease of the dielectric constant of the solvent, and incubation in those conditions for up to four days, results in modest ThT and ANS fluorescence increase when compared to the results obtained for mature fibrils after 60 days of incubation. On the other hand, this change of the solvent almost immediately results in prominent qualitative changes of FTIR spectra. While the Amide II region shows only a slight shift, as it is less dependent on the secondary structure content, the Amide I and Amide III regions change completely in their shapes. Both regions provide proves for the shift towards β-sheet bands, on the count of α-helix and random coil upon transition to ethanol solution. More sensitive Amide I region has the additional advantage of differentiation between intermolecular and intramolecular β-sheet and offers the possibility of quantitative monitoring of structural changes. The appearance of aggregation specific β-sheet band in the Amide I region is evidence of HEWL propensity to form ordered aggregates.

Deconvoluted Amide I region of FTIR spectra shows that oligomer state, stable during the first four days of HEWL incubation in ethanol-rich conditions, is structurally closer to mature fibril state obtained by 60-day incubation in mentioned conditions than to original native monomer. Nevertheless, AFM microscopy, as a suitable replacement for oligomer-specific antibody binding, proves that HEWL is still in this intermediate oligomer state after four days of incubation. Drastic secondary structure perturbations in oligomer compared to monomer state, namely 20% α-helix loss and about 26% aggregation-specific β-sheet increase, does not change strongly during the rest of the fibrillation process (mature fibrils show 22% of total α-helix loss and about 30% of the aggregation-specific β-sheet increase). 

Unlike precise but complicated deconvolution methods that meet limitations in analyzing biological samples, the ratio of the Amide I bands to the Amide II internal standard provides a fast and simple method for monitoring the propensity of the secondary structure change. Applying this method shows a significant increase of intramolecular and significant decrease of nativelike secondary structures that can be used as a simple quantitative method for oligomers detection. It can be easily applied to biological samples with high protein concentrations, such as liquor, and provide a fast screening method for these toxic species.

## Figures and Tables

**Table 1:** Secondary structure content derived from HEWL samples by the deconvolution of attenuated total reflectance—Fourier transform infrared spectroscopy (ATR-FTIR) spectra in the Amide I region and literature data derived from X-ray diffraction [11].

HEWL Sample	β-Sheet	Aggregation β-Sheet	α-Helix	Random Coil + Turn	Error
native, H_2_O	10.3	8.6	41.7	39.4	1
0 h, 90% ethanol	10.5	29.7	21.8	38	0.8
24 h, 90% ethanol	10.1	34.7	22.8	32.4	0.6
96 h, 90% ethanol	10.2	35.1	21.8	32.9	1.1
60 days, 90% ethanol	10.3	38.2	19.6	31.9	0.8
X-ray diffraction	18.7	/	42.6	38.7	/

## Data Availability

Data is contained within the article and Supplementary Material.

## Supplementary Materials

The following are available online. Table S1. Amide I/Amide II ratio for the quantitative assessment of oligomer formation. Figure S1. Correlation of ThT fluorescence with normalized aggregation β-sheet IR absorbance of HEWL samples during 4-day incubation.

## Sample Availability

Samples of HEWL oligomers are available from the authors.
