# Supplementary material for: On the Protein Fibrillation Pathway: Oligomer Intermediates Detection Using ATR-FTIR Spectroscopy
Source: Molecules. 2021 Feb 12;26(4):970. doi: 10.3390/molecules26040970 (PMC7918411; doi:10.3390/molecules26040970)
Supplement: Supplementary file 1 [file molecules-26-00970-s001.pdf]

Table S1. Amide I / Amide II ratio for the quantitative assessment of oligomer formation. Values at given wavenumbers in the Amide I region are normalized to 1557 cm^{-1} band in the Amide II region.

**Sample**	**α-helix (1655 cm-1)**	**Random coil (1645 cm-1)**	**β-sheet (1622 cm^{-1})**	**β-sheet (1698 cm^{-1})**
native HEWL	3.10	2.93	1.10	0.66
0 h	1.93	1.90	2.14	0.72
1 h	1.86	1.85	2.11	0.68
2 h	1.86	1.84	2.11	0.70
4 h	1.94	1.90	2.14	0.74
8 h	1.84	1.81	2.10	0.70
16 h	1.88	1.85	2.11	0.65
24 h	1.99	1.95	2.18	0.76
48 h	1.94	1.89	2.12	0.73
72 h	1.88	1.92	2.10	0.76
96 h	1.98	1.93	2.19	0.78

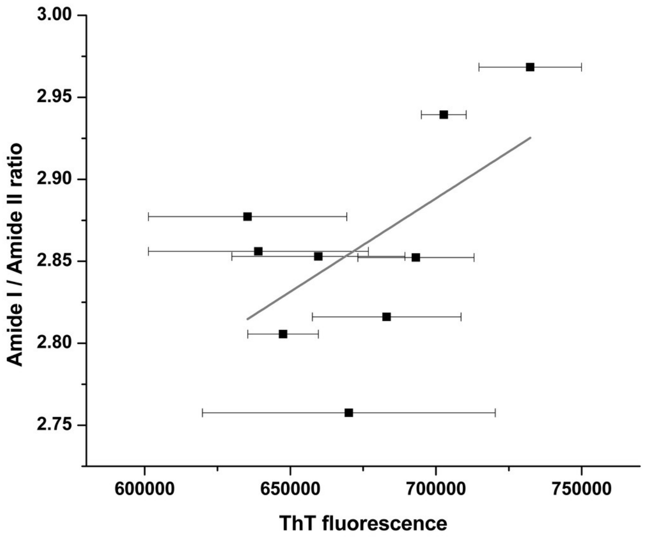

Figure S1. Correlation of ThT fluorescence with normalized aggregation β-sheet IR absorbance of HEWL samples during 4-day incubation.
